# Determinants of flammability in savanna grass species

**DOI:** 10.1111/1365-2745.12503

**Published:** 2015-11-26

**Authors:** Kimberley J. Simpson, Brad S. Ripley, Pascal‐Antoine Christin, Claire M. Belcher, Caroline E. R. Lehmann, Gavin H. Thomas, Colin P. Osborne

**Affiliations:** ^1^ Department of Animal and Plant SciencesUniversity of SheffieldSheffieldS10 2TNUK; ^2^ Department of BotanyRhodes UniversityPO Box 94Grahamstown6140South Africa; ^3^ College of Life and Environmental SciencesUniversity of ExeterExeterEX4 4PSUK; ^4^ School of GeoSciencesUniversity of EdinburghEdinburghEH9 3JNUK

**Keywords:** biomass moisture content, biomass quantity, determinants of plant community diversity and structure, fire regime, functional traits, phylogeny, poaceae, resprouting

## Abstract

1. Tropical grasses fuel the majority of fires on Earth. In fire‐prone landscapes, enhanced flammability may be adaptive for grasses via the maintenance of an open canopy and an increase in spatiotemporal opportunities for recruitment and regeneration. In addition, by burning intensely but briefly, high flammability may protect resprouting buds from lethal temperatures. Despite these potential benefits of high flammability to fire‐prone grasses, variation in flammability among grass species, and how trait differences underpin this variation, remains unknown.
2. By burning leaves and plant parts, we experimentally determined how five plant traits (biomass quantity, biomass density, biomass moisture content, leaf surface‐area‐to‐volume ratio and leaf effective heat of combustion) combined to determine the three components of flammability (ignitability, sustainability and combustibility) at the leaf and plant scales in 25 grass species of fire‐prone South African grasslands at a time of peak fire occurrence. The influence of evolutionary history on flammability was assessed based on a phylogeny built here for the study species.
3. Grass species differed significantly in all components of flammability. Accounting for evolutionary history helped to explain patterns in leaf‐scale combustibility and sustainability. The five measured plant traits predicted components of flammability, particularly leaf ignitability and plant combustibility in which 70% and 58% of variation, respectively, could be explained by a combination of the traits. Total above‐ground biomass was a key driver of combustibility and sustainability with high biomass species burning more intensely and for longer, and producing the highest predicted fire spread rates. Moisture content was the main influence on ignitability, where species with higher moisture contents took longer to ignite and once alight burnt at a slower rate. Biomass density, leaf surface‐area‐to‐volume ratio and leaf effective heat of combustion were weaker predictors of flammability components.
4. *Synthesis*. We demonstrate that grass flammability is predicted from easily measurable plant functional traits and is influenced by evolutionary history with some components showing phylogenetic signal. Grasses are not homogenous fuels to fire. Rather, species differ in functional traits that in turn demonstrably influence flammability. This diversity is consistent with the idea that flammability may be an adaptive trait for grasses of fire‐prone ecosystems.

## Introduction

Fire is a disturbance that has shaped plant traits and floral communities for over 420 million years (Glasspool, Edwards & Axe 2004; Bond, Woodward & Midgley 2005) and acts as a powerful selective filter for functional traits related to plant persistence, recovery and recruitment (Emerson & Gillespie 2008). Fire is also multidimensional and its effects on vegetation depend on the characteristics of the local fire regime (Keeley *et al*. 2011), which can vary considerably in frequency, intensity, size and season (Archibald *et al*. 2013). Different fire regimes can lead to the assembly of distinct populations and communities that are functionally clustered for diverse traits (Pausas & Bradstock 2007; Verdú & Pausas 2007; Silva & Batalha 2010; Forrestel, Donoghue & Smith 2014). For example, resprouting species are favoured in frequent, low‐intensity fire regimes, and obligate seeders that persist via seedling recruitment are favoured in infrequent, high‐intensity fire regimes (Pausas & Bradstock 2007; Pausas & Keeley 2014).

Plant flammability may both influence and be influenced by fire regime (He, Lamont & Downes 2011; Pausas *et al*. 2012) but species variation in flammability has received relatively little attention (but see Scarff & Westoby 2006; Murray, Hardstaff & Phillips 2013; Grootemaat *et al*. 2015). Flammability is an emergent property of a plant's chemical and physical traits. However, the identification of these traits in several fire‐prone taxa, particularly herbaceous species, has not been achieved. Flammability as a vegetation property consists of several interdependent components (Anderson 1970) that can each be quantified. Ignitability (the ease of ignition), combustibility (the intensity of combustion) and sustainability (the maintenance of burning over time) are flammability components and can be measured at multiple scales. For example, ignitability is often measured as ignition delay at the leaf or plant scale, while the rate of fire spread is a measure of ignitability that operates at the community scale (Gill & Zylstra 2005).

Plant flammability is a key determinant of fire behaviour (Bond & van Wilgen 1996; Beckage, Platt & Gross 2009). In woody plants, flammability varies considerably between and within species (e.g. Fonda 2001; Saura‐Mas *et al*. 2010; Pausas *et al*. 2012; Cornwell *et al*. 2015), and minor changes in vegetation composition have repeatedly demonstrated significant alterations in vegetation flammability and fire regime (Rossiter *et al*. 2003; Brooks *et al*. 2004; Belcher *et al*. 2010). Flammability may act as a means by which plants modify fire regimes to engender favourable conditions (Schwilk 2003). For example, slow‐growing, woody, obligate seeder species, such as *Pinus* species, require infrequent intense fire to complete their life cycle. High‐temperature crown fires are vital for releasing stored seeds from the retained mature cones of these serotinous species and enhancing recruitment opportunities of seedlings via mortality of neighbouring trees (Lamont *et al*. 1991; Keeley *et al*. 2011). In contrast, resprouting perennial grasses, which dominate grasslands and savannas (Uys 2000; Allan & Southgate 2002; Overbeck & Pfadenhauer 2007), may benefit from very frequent fire (Archibald *et al*. 2013). These shade‐intolerant species require the regular removal of standing dead biomass (Everson, Everson & Tainton 1988) and woody growth (Bond 2008), which may be aided by high plant flammability. Surface fires in grassy systems are characterized by rapid combustion and spread, low fire residence times and cool burn temperatures (Bradstock & Auld 1995; Archibald *et al*. 2013). Such fire characteristics are advantageous to resprouting grass species, protecting basal meristems from excessive heat through biomass that burns rapidly (Gagnon *et al*. 2010). In addition, high flammability, if linked to efficient post‐fire recovery, may provide enhanced regeneration opportunities for these species by killing neighbouring plants and reducing post‐fire competition (Bond & Midgley 1995).

Despite these predicted benefits of frequent fire to fire‐prone grasses, interspecific variation in the flammability of such species has been little explored (Ripley *et al*. 2010), in contrast to knowledge about interspecific variation in post‐fire response among grass species (Ripley *et al*. 2015). A historical belief persists that grasses and other herbaceous plants vary little in their flammability, which has led to the diversity of herbaceous fuels being reduced to one or few fuel classes in fire behaviour modelling (e.g. Anderson 1982). Given the considerable known variation in the flammability of woody species (Schwilk 2003; Scarff & Westoby 2006; Pausas *et al*. 2012; Murray, Hardstaff & Phillips 2013), such presumptions are unfounded. Substantial changes in grassland community flammability resulting from invasion by non‐native grasses provide evidence to suggest considerable interspecific variation in grass flammability (Hughes, Vitousek & Tunison 1991; Rossiter *et al*. 2003). In addition, recent evidence shows that grass traits relating to post‐fire recovery are shaped by fire regime (Forrestel, Donoghue & Smith 2014; Ripley *et al*. 2015), suggesting that traits relating to flammability may be responding in similar ways, resulting in intra‐ and interspecific variation in flammability.

Physical and chemical traits influencing some or all components of flammability relate to the quantity, quality, moisture content and aeration of biomass (Bond & van Wilgen 1996; Gill & Moore 1996). Biomass quantity is critical to combustibility and fire spread rate because it directly influences fire energy output rate (Byram 1959; Rothermel 1972). Biomass moisture content determines the extent to which fuels absorb heat energy, with high values associated with delayed ignition and low combustion and fire spread rates (Pyne 1984; Nelson 2001). Biomass surface‐area‐to‐volume (SA/V) ratio influences curing and reaction rates within fires (Papio & Trabaud 1991; Gill & Moore 1996), with high values linked to rapid ignition, and rapid rates of combustion and fire spread. Increasing biomass density, defined as the mass of biomass per unit volume of fuel bed, raises fuel connectivity, therefore enhancing combustibility and fire spread rate. This relationship applies up to a certain threshold beyond which poor ventilation will limit drying and combustion rates (Rothermel 1972). Intrinsic properties of plant material, such as heat of combustion, affect combustibility and fire spread rate through the amount of heat energy released during complete combustion. Sustainability is often inversely related to combustibility and ignitability (e.g. de Magalhães & Schwilk 2012). Therefore, plant traits likely to enhance combustion and spread rate may indirectly reduce flaming duration. In contrast, high biomass quantity increases combustion and spread, but is also likely to enhance sustainability, as more fuel takes longer to burn. Plant traits important to flammability have been identified in a number of fire‐prone taxa (e.g. Ganteaume *et al*. 2013; Schwilk & Caprio 2011). However, the traits that influence grass flammability, and more generally the flammability of herbaceous species, have not been empirically established or explored.

We examined three components of flammability, at multiple scales, for 25 species common in fire‐prone South African grasslands. Five structural and chemical plant traits, known to influence vegetation flammability, were measured and correlated with flammability trait values (see Table 1). We hypothesized that (i) there is significant interspecific variation in flammability among grass species and that (ii) the measured plant traits can explain this variation, with each trait contributing to flammability components in different ways (see Table 1 for specific predictions). We also expected that flammability and plant traits covary due to the interdependent relationships between flammability components and plant traits. The strong phylogenetic patterns in grass distributions across fire‐frequency gradients (e.g. Visser *et al*. 2012; Forrestel, Donoghue & Smith 2014) led us to predict that (iii) flammability is influenced by evolutionary history and contains a phylogenetic signal.

**Table 1:** Matrix summarizing the predicted relationships between plant and flammability traits. Flammability traits were determined at different scales (L, leaf; P, plant; C, community) and represent three flammability components. Symbols reflect the direction of the relationship (‘+’: positive; ‘−’: negative; ‘0’: none; ‘N/A’: could not be tested). Influence is either direct or indirect (in parentheses)

			Plant trait				
Flammability trait	Flammability component	Scale	Biomass quantity (g)	Biomass density (g cm^−1^)	Biomass moisture content (g g^−1^)	Leaf SA/V ratio	Leaf effective heat of combustion (J g^−1^)
Time to ignition (s)	Ignitability	L	N/A	N/A	−	+	0
Predicted rate of fire spread (m s^−1^)	Ignitability	C	+	+	−	+	+
Flaming time (s)	Sustainability	L, P	+	(−)	(+)	(−)	(−)
Combustion rate (g s^−1^)	Combustibility	L, P	+	+	−	+	+

## Materials and methods

### Plant Material

Plants were collected during the natural fire season in July 2014 in grassland and Nama‐Karoo habitats near Grahamstown in the Eastern Cape of South Africa (see Table S1 in Supporting Information for site details). Fire return times over the 2000–2006 period were 2.3 years for vegetation surrounding Grahamstown (Tansey *et al*. 2008).

Seven individuals of 25 species, representing 5 grass subfamilies, were collected for study (see Table S2). All species were native to the region except *Cenchrus setaceus*, a North African invasive species (Milton 2004). For each species, seven randomly selected, healthy‐looking adult plants were dug up while keeping their shoot architecture intact. Plants were stored in sealed plastic bags at room temperature for a maximum of 48 h to minimize changes in moisture content. A specimen of each species was deposited at the Selmar Schonland Herbarium (Rhodes University).

### Structural and Chemical Traits

A section of each individual (approximately one‐third of the entire plant), with its below‐ground biomass and soil removed, was used to measure five structural and chemical plant traits. Biomass quantity, density and moisture content were measured at the plant scale, while effective heat of combustion (EHoC) and SA/V ratio were measured at the leaf scale.

For measurements of leaf SA/V ratio and EHoC, leaves were removed from a randomly selected tiller of each individual. Total leaf area was measured on digital images using the computer program WinDIAS (Delta‐T Devices, Cambridge, U.K.) that determines leaf area by selecting pixels of a pre‐defined colour range. Leaf thickness was measured, at the middle of the leaf and excluding the midrib, for three leaves per tiller using digital callipers (accurate to 0.01 mm), and an average value was calculated. Leaf SA/V ratio was calculated from the average leaf area and leaf thickness of each species.

The heat of combustion is the energy released as heat when biomass undergoes complete combustion with oxygen, which typically relates to C:N ratio, lignin content and the presence of flammable compounds (Philpot 1969; Bond & van Wilgen 1996). We measured the EHoC, which is the heat of combustion of pyrolysate vapours, and does not assume that all char is consumed. Compared to measurements that involve the full thermal decomposition of biomass (such as in bomb calorimetry), EHoC is a more realistic estimate of the energy released from a wildfire in which combustion is incomplete, and most of the energy is released from burning the pyrolysate vapours. Oven‐dried leaf samples of known mass (5.0 ± 0.4 mg) were conditioned at room temperature and humidity before being analysed in a microscale combustion calorimeter following the manufacturer's guidelines (FAA Micro Calorimeter, Fire Testing Technology Ltd, East Grinstead, UK). Each sample was held in nitrogen and heated at a rate of 3 °C per second driving off the volatile gases that were ignited and completely oxidized, and heat release was quantified by oxygen depletion calorimetry (Tewarson 2002). Total heat release was divided by the sample mass to provide the EHoC (kJ g^−1^). Due to the high repeatability of this trait measurement, material from three randomly chosen individuals per species was tested in duplicate, to give an average value per individual and per species.

For plant‐scale traits, the height (maximum vertical distance from ground level to the tallest point) and width (maximum horizontal spread) of each clump was determined. Biomass density was measured using a novel method, which determined the vertical biomass distribution for each individual. For this, the biomass of each clump was divided at five or more equal intervals along its vertical height, so that intervals were 2.5, 5, 10 or 15 cm in length depending on the plant height, and started at ground level. Each clump was cut with scissors at the selected intervals. The fresh and dry biomass of each section were weighed to four decimal places, the latter after oven drying at 70 °C to a constant weight. Cumulative dry biomass was calculated at each vertical height interval from ground level. Linear models were fitted to the logged cumulative dry biomass and vertical height for each individual. The slope of this relationship was used as a proxy for biomass density, in g cm^−1^, with high values indicating densely packed biomass. For each clump, dry biomass values were combined to give the total dry biomass, and moisture content was calculated by dividing the difference between fresh and dry biomass by the dry biomass.

### Flammability

Flammability was represented by three components: ignitability, combustibility and sustainability (Anderson 1970). All components were measured for each individual at the leaf scale via epiradiator tests. In addition, combustibility and sustainability were determined at the plant scale by burning partial plant canopies. Plant‐scale measurement of ignitability was beyond the scope of this experiment; however, a community‐level measure was obtained by estimating the rate of fire spread for each individual by parameterizing Rothermel's (1972) fire spread model with plant trait data. Leaf‐ and plant‐scale flammability components were measured both on fresh and dry biomass to determine the effect of moisture content. The ‘fresh’ clump was kept in a sealed plastic bag at room temperature, and the ‘dry’ clump was first dried at 70 °C for a minimum of 48 h.

Leaf‐scale ignitability, sustainability and combustibility were measured as time to ignition, flaming time and mass loss rate, respectively, using a Quartz infrared 500 W epiradiator (Helios, Italquartz, Milan, Italy) in a fume cupboard with a constant vertical windspeed of 0.1 m s^−1^. As application of leaf material directly to the epiradiator's silica disc surface always caused instantaneous combustion, 2‐mm wire mesh was positioned 1 cm above the epiradiator's surface. The background temperature at the mesh surface (without fuel), measured by a thermocouple connected to a data‐logger, ranged between 370 and 400 °C. Samples of 0.2 g (±0.001 g) leaf material were cut into 2‐cm segments to standardize between samples and applied to the centre of the mesh. The 0.2 g mass was used because preliminary studies found that smaller masses failed to ignite, while larger fuel masses increased the risk that other fuel properties, particularly fuel height, influenced flammability values. Smaller samples were used for *Aristida congesta* subsp. *barbicollis* due to the low leaf mass of this species. Each test was filmed at 25 frames s^−1^, and (i) time to ignition (TTI; the time between sample application to the epiradiator and first flaming) and (ii) flaming time (FT; the time from ignition to flame extinction) were subsequently determined. As samples were completely combusted by applying them to the epiradiator, an average leaf combustion rate was obtained by dividing the mass of samples by FT. Species average values for TTI and FT were obtained for fresh and dry material. The influence of leaf moisture content on these flammability traits was determined as the difference in values between fresh and dry samples of each individual and averaged per species.

As canopy architecture influences grass flammability (Martin 2010), a method that measures plant‐scale flammability traits was utilized. Fresh and dry plant material from each individual were clamped on a stand on a four‐point balance (Mark 205A; Bel Engineering, Monza, Italy) and burnt in a fume cupboard with a constant 0.1 m s^−1^ vertical wind speed (see Figure S1 for diagram of the set‐up used). Samples were ignited by directing a Bunsen burner flame to the side of the base of the clump at a 45° angle and a 5 cm distance for a maximum of 3 s (less if ignition happened earlier). This resulted in successful ignition in all individuals. Mass loss was logged at 0.2‐s intervals and the sigmoidal relationship produced was fitted with a Boltzmann equation. Data were excluded if fitting the relationship was not possible due to noise around the curve (*n* = 40/350), which occurred if large pieces of plant material fell off the balance during a burn. The width parameter used to fit the Boltzmann curve reflects the time period in which mass was drastically reduced and was used as a plant‐scale measurement of sustainability (flaming time). Three seconds of data either side of the inflection point were selected and a linear regression fitted. The slope of this regression represents the maximum combustion rate in g s^−1^. As preliminary results found this combustibility trait to be strongly driven by the biomass of the sample, interspecific comparisons were standardized for mass. Therefore, maximum combustion rate was plotted against mass change for each species, and linear models were fitted to the fresh, dry and combined data sets. As there was no change in mass common to all 25 species, the y‐intercept extracted from the model fitted to the combined data set was used to characterize the intrinsic combustibility of each species. The combined data set was used as the slopes of the models fitted to the fresh and dry data did not differ significantly for any species, and model fit was improved by combining the data sets. Any unpaired samples were excluded to ensure a balanced data set of fresh and dry samples. The y‐intercept differed significantly between fresh and dry models for three species (*Panicum* sp., *Hyparrhenia hirta* and *Merxmuellera stricta*) and in these cases, the y‐intercept was extracted from linear models fitted to the fresh data set.

Forward fire spread rate values, the community‐scale measure of ignitability, were predicted for each individual using Rothermel's (1972) surface fire spread model as implemented using the ros() function in the *Rothermel* package (Vacchiano & Ascoli 2014) in R (R Core Team 2013). Fire behaviour was simulated for each individual by parameterizing the model with data for the following traits: leaf SA/V ratio, leaf EHoC, biomass moisture content, plant height and fuel load (biomass quantity divided by the estimated cover area). See Table S3 for a details of the procedure followed and model assumptions.

### Phylogenetic Analysis

We constructed a phylogeny that was initially based on a previously generated data set for grasses composed of the plastid markers *trnKmatK*,* ndhF* and *rbcL* (Grass Phylogeny Working Group II 2012) and augmented here. For ten species not represented in this previous data set, a fragment of *trnKmatK* was PCR‐amplified from genomic DNA, following protocols and primers described previously (Grass Phylogeny Working Group II 2012). The newly generated sequences have been submitted to NCBI database (Benson *et al*. 2012) under the accession numbers KP860326 to KP860336. The new markers were manually aligned to the data set, which consisted of 606 taxa and 5649 aligned bp. This initial data set was downsized to 70 species, including all the taxa studied here and representatives of all grass lineages. A time‐calibrated phylogenetic tree was obtained through Bayesian inference as implemented in BEAST (Bayesian evolutionary analysis by sampling trees; Drummond & Rambaut 2007). A general time‐reversible substitution model with a gamma‐shape parameter and a proportion of invariants (GTR+G+I) were used. The log‐normal relaxed clock was selected. The tree prior was modelled by a Yule process. The monophyly of the BEP‐PACMAD clade was enforced, leaving *Puelia olyriformis* as the outgroup. The calibration prior for the age of the BEP‐PACMAD crown was set to a normal distribution, with a mean of 51.2 and a standard deviation of 0.001 (mean based on Christin *et al*. 2014). Two independent runs were conducted for 10 000 000 generations, sampling a tree every 1000 generations. The convergence of the runs and the appropriateness of the burn‐in period, set to 2 000 000 generations, were verified using Tracer (Rambaut A, Drummond AJ (2007) Tracer v1.4, available at http://beast.bio.ed.ac.uk/Tracer). Median ages were mapped on the maximum‐credibility tree. The relationships among the species studied here were extracted from this tree and used for comparative analyses.

### Data Analysis

Statistical analyses were carried out in the R environment (R Core Team 2013). Data were log‐transformed to improve normality and to meet model assumptions where necessary.

Analysis of variance (anova) was used to determine whether plant and flammability traits differed significantly between species. The influence of species, and state (‘fresh’ or ‘dry’), on leaf‐scale flammability was determined by two‐way anova. As biomass quantity values for the plant‐scale burns are not representative of the species (i.e. for each species, clumps were subsampled and a range of masses were burnt), a species effect on the relationship between maximum combustion rate and biomass quantity was tested using the R package MCMCglmm (Hadfield 2010). This approach implements Markov chain Monte Carlo routines for fitting generalized linear mixed models, while accounting for non‐independence and correlated random effects arising from phylogenetic relationships (Hadfield 2010). We fitted flammability (maximum combustion rate) and biomass quantity as a bivariate normal response, and species as a random effect. Models were run for 500 000 iterations with a burn‐in of 1000 iterations, a thinning interval of 500 and weakly‐informative priors (*V* = diag(2), nu = 0.002). The 95% highest posterior densities (HPD) of within‐species and across‐species slopes and the difference between slopes were estimated while accounting for phylogeny and used to assess whether slopes differed among species.

To test the hypotheses put forward in Table 1 and to establish the strength and direction of plant trait contributions to flammability components, a MCMC multi‐response generalized linear mixed model approach was used again. Traits were separated into leaf and plant scale to ensure appropriate comparisons, using the same prior and specifications as before. The fit of the models to data was established by fitting linear models between the observed flammability trait values and those predicted by the models. The contribution of plant traits to fire spread rate was tested to determine whether strong relationships occurred across species when accounting for phylogeny, while acknowledging that some circularity is involved because spread rate was predicted based on the values of these traits.

To explore the pattern of covariance among plant and flammability traits, principal component analyses were performed using the princomp function (R core team 2013). Linear regressions were also used to establish the relationships among plant and flammability traits, with the latter being split into leaf‐scale and plant‐scale traits for analyses to ensure an appropriate comparison. The relationships between flammability traits measured at different scales were also established using linear regressions.

The influence of evolutionary history was established for each plant and flammability trait by testing for the presence of a phylogenetic signal. This was done using the pgls function in the *caper* package (Orme *et al*. 2012) which estimated Pagel's λ.

## Results

### Flammability Variation Among Species

**Figure 1:**
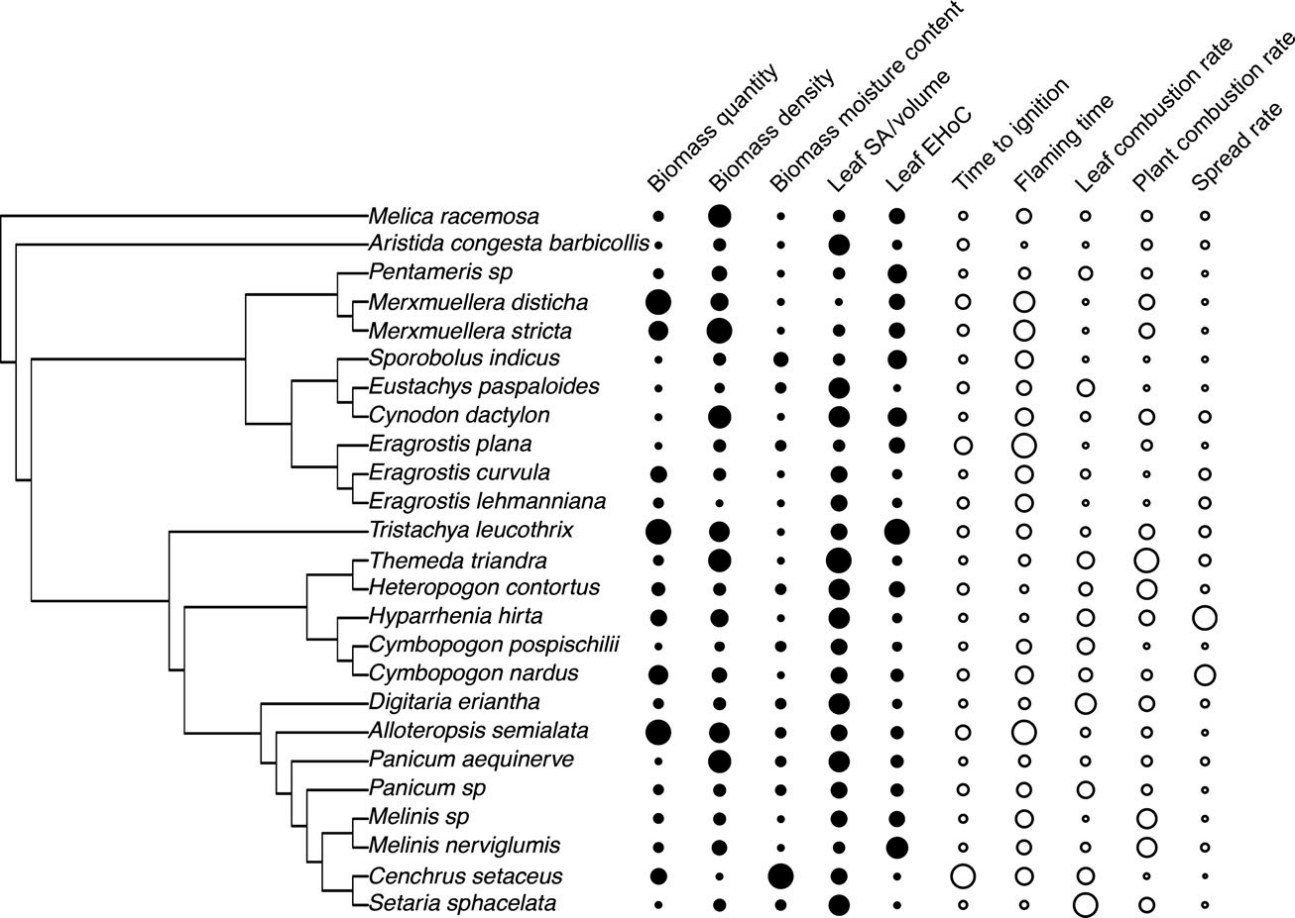
The evolutionary relationships between species and average values of explanatory plant traits (solid circles) and flammability traits (open circles). Trait values are indicated by the size of the circles. A nonzero phylogenetic signal was found for leaf SA/V ratio (Pagel's λ = 1; *P *=* *1 for λ = 1; *P *<* *0.001 for λ = 0), leaf flaming time (Pagel's λ = 0.45; *P *=* *1.0 for λ = 1; *P *<* *0.001 for λ = 0) and leaf combustion rate (Pagel's λ = 0.99; *P *=* *0.93 for λ = 1; *P *=* *0.037 for λ = 0).

All flammability components varied considerably across species (Fig. 1; Table S4). At the leaf‐scale, significant interspecific variation was found in ignitability (*F*_24,144_ = 5.02, *P *< 0.001), sustainability (*F*_24,144_ = 3.02, *P *< 0.001) and combustibility (*F*_24,144_ = 2.97, *P *< 0.001). Ignition delays ranged from 1.0 s (*H. hirta*) to 4.0 s (*C. setaceus*) with a mean across species of 1.7 s. The mean flaming duration across species was 6.3 s and ranged from 4.3 s (*A. congesta* subsp. *barbicollis*) to 7.6 s (*Eragrostis plana*). Connected to flaming duration was average combustion rate, with *E. plana* burning at the slowest rate (27 mg s^−1^) and *A. congesta* subsp. *barbicollis* at the fastest (49 mg s^−1^).

**Figure 2:**
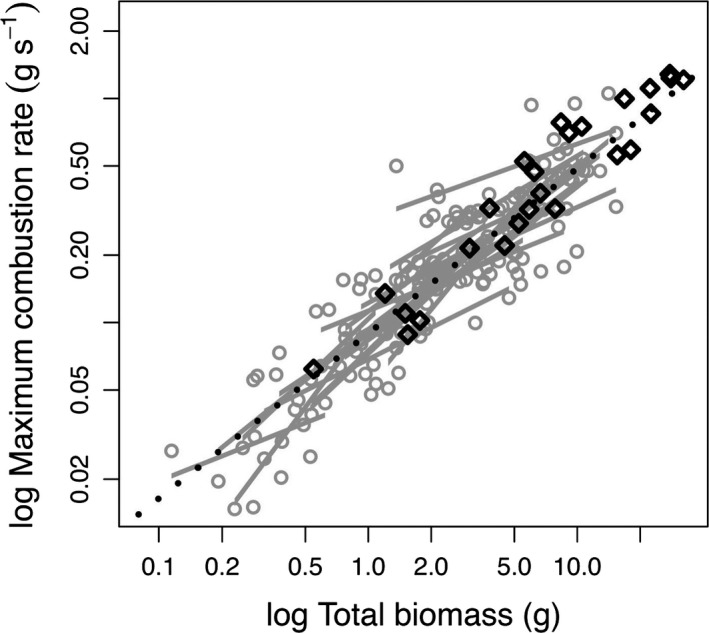
Relationships between biomass quantity and maximum combustion rate across 25 grass species. The mean slopes of within‐species relationships (grey lines) and across‐species relationships (black dotted line) for maximum combustion rate with biomass burned do not differ significantly when phylogeny is accounted for. Data points are shown as grey circles. Estimates of whole‐plant combustion rates (black diamonds) showed substantial variation (>20‐fold). These values were calculated by extrapolating the common across‐species relationship (black dashed line) to species mean total biomass values while taking into account the intrinsic combustibility differences among species.

At the plant scale, intrinsic combustibility (for a given biomass) differed by <2.5‐fold across species, ranging from 0.064 g s^−1^ (*Eustachys paspaloides*) to 0.163 g s^−1^ (*Themeda triandra*). When investigating the relationship between combustion rate and biomass, the bivariate mixed effects model revealed that within‐species slopes (pooled mean = 0.594, HPD: 0.507 to 0.707) and across‐species slopes (mean = 0.797, HPD: 0.067 to 1.385) did not differ significantly (mean slope difference (Δb) = 0.212, HPD: −0.521 to 0.683) when accounting for phylogeny (Fig. 2). This common relationship was extrapolated while taking into account intrinsic combustibility differences, allowing combustion values to be predicted for the species mean total biomass. These predicted values of whole‐plant combustion rates varied >20‐fold among species, ranging from 0.06 g s^−1^ (*A. congesta* subsp. *barbicollis*) to 1.28 g s^−1^ (*M. disticha*; Fig. 2).

Fuel models based on the traits of *C. setaceus* predicted no fire spread, because biomass moisture content values exceeded the moisture of extinction, defined as the fuel moisture content above which a steady rate of fire spread is not possible. Of the remaining species that spread fire, the estimated rate of spread differed substantially (25‐fold; Table S4) and varied significantly between species (anova:* F*_24,150_ = 42.42, *P *<* *0.001).

Substantial interspecific variation was also found in the five traits measured as explanatory traits for flammability (Fig. 1; see Table S5). Biomass moisture content values of the non‐native *C. setaceus* were substantially higher than the other species. However, species still differed significantly for this trait when *C. setaceus* was excluded (anova:* F*_23,144_ = 14.39, *P *<* *0.001). The measurement of biomass density (i.e. vertical biomass distribution) produced consistent values within species (Fig. S2; species average CV = 28%), but considerable differences among species with slope values ranging from 0.155 (*Eragrostis lehmanniana*) to 0.831 (*M. stricta*).

Collection site did not influence flammability traits. Of the plant traits, vertical biomass distribution (*P *=* *0.008) and leaf EHoC (*P *=* *0.046) were the only ones affected by collection site (see Table S7).

### Trait Contributions to Flammability

Measured plant traits significantly predicted the components of flammability, particularly ignitability and plant‐scale combustibility, in which 70% and 58% of variation could be explained by the plant traits, respectively (Tables 2 and 3). Variation in sustainability could be explained to a lesser extent by plant traits at the leaf (47%) and plant scale (37%), as well as variation in leaf‐scale combustibility (39%). The direction of relationships between plant and flammability traits is consistent with those predicted in Table 1, but there are exceptions. Both biomass density and leaf SA/V ratio were expected to correlate positively with predicted spread rate, but instead correlated negatively (Table 3).

**Table 2:** The contribution of plant traits to leaf‐scale flammability components as determined by MCMC phylogenetic generalized linear mixed models. Values represent posterior mean estimates of the slopes, the upper and lower 95% confidence intervals and *P* values (those in bold are significant at *P *=* *0.05). In combination, species mean trait values of leaf moisture content, SA/V ratio and effective heat of combustion (EHoC) significantly predicted ignitability (*F*_1,166_ = 398.3, *P *<* *0.001, *R* ^2^ = 0.70), sustainability (*F*_1,166_ = 147.5, *P *<* *0.001, *R* ^2^ = 0.47) and combustibility (*F*_1,166_ = 105.4 *P *<* *0.001, *R* ^2^ = 0.39)

		Leaf moisture content^a^	Leaf SA/V ratio	log Leaf EHoC
Ignitability (time to ignition)	Estimate	0.691	−0.174e‐3	−0.135e‐4
	(95% CI)	(0.620 to 0.760)	(−0.420e‐3 to 0.872 e‐5)	(−0.527e‐4 to 0.290e‐4)
	*P* value	**<0.001**	0.17	0.49
Sustainability (flaming time)	Estimate	0.492	−0.876e‐3	0.159e‐4
	(95% CI)	(0.421 to 0.567)	(−0.142e‐2 to ‐0.359 e‐4)	(−0.626e‐4 to 0.113e‐3)
	*P* value	**<0.001**	**0.002**	0.741
Combustibility (combustion rate)	Estimate	−0.303e‐2	0.522e‐5	−0.227e‐6
	(95% CI)	(−0.406e‐2 to −0.170e‐2)	(−0.547e‐5 to 0.164e‐4)	(−0.254e‐5 to 0.193e‐5)
	*P* value	**<0.001**	0.36	0.86

a Parameter characterized as: the species mean difference in ignition delay (for ignitability) or flaming duration (for sustainability and combustibility) between fresh and dry leaf material for each individual.

**Table 3:** The contribution of plant traits to plant‐scale flammability components as determined by MCMC phylogenetic generalized linear mixed models. Values represent posterior mean estimates of the slopes, the upper and lower 95% confidence intervals and *P* values (those in bold are significant at *P *=* *0.05). Values represent posterior mean estimates of the slopes, the upper and lower 95% confidence intervals and *P* values (those in bold are significant at *P *=* *0.05). In combination, the five plant traits significantly predicted sustainability (*F*_1,151_ = 90.07, *P *<* *0.001, *R* ^2^ = 0.37), combustibility (*F*_1,151_ = 210.8, *P *<* *0.001, *R* ^2^ = 0.58) and ignitability (*F*_1,173_ = 184.2, *P *<* *0.001, *R* ^2^ = 0.51)

		log Biomass quantity	log Biomass density	log Biomass moisture content	Leaf SA/V ratio	log Leaf EHoC^a^
Sustainability (flaming time)	Estimate	0.434	−0.614	1.036	−0.050	−0.012
	(95% CI)	(0.350 to 0.517)	(−2.162 to 0.889)	(−0.688 to 2.753)	(−0.162 to 0.055)	(−0.023 to 0.001)
	*P* value	**<0.001**	0.443	0.252	0.363	0.060
Combustibility (maximum combustion rate)	Estimate	0.035	0.149	−0.108	0.105e‐2	−0.580e‐4
	(95% CI)	(0.028 to 0.041)	(0.021 to 0.277)	(−0.250 to 0.027)	(−0.858e‐2 to 0.012)	(−0.101e‐2 to 0.103e‐2)
	*P* value	**<0.001**	**0.024**	0.116	0.910	0.826
Ignitability (predicted spread rate)	Estimate	2.002	−0.061	−0.034	0.128e‐2	0.121e‐3
	(95% CI)	(0.951 to 3.015)	(−0.094 to −0.033)	(−0.044 to −0.025)	(0.789e3 to 0.169e‐2)	(−0.993e‐4 to 0.360e‐3)
	*P* value	**<0.001**	**<0.001**	**<0.001**	**<0.001**	0.309

a Species mean values.

Moisture content was key in determining leaf‐scale flammability components (Table 2; Table S6). Ignitability was particularly influenced by moisture content, with fresh leaf material taking 42% longer to ignite on average than dry leaf material across species, with a maximum increase of 288% seen for *C. setaceus* (1.0 s dry vs. 4.0 s fresh). Once alight, fresh leaf material also burned on average for 7% longer at a 3% lower combustion rate compared to dry leaf material across species. Leaf SA/V ratio significantly influenced sustainability, with high values associated with low flaming duration. The EHoC of leaf material alone contributed little to overall leaf‐scale flammability when compared to moisture or SA/V ratio (Table 2).

At the plant scale, biomass quantity was by far the strongest driver of sustainability and combustibility (Table 3). Plants with greater biomass burnt at a faster rate and for longer. Biomass density and moisture content significantly contributed to plant‐scale combustibility, such that plants with high density and low moisture content combusted most rapidly (Table 3). The EHoC of leaf material significantly contributed to sustainability with high values associated with short flaming times (Table 3). Leaf SA/V ratio did not significantly contribute to plant‐scale combustibility or sustainability.

Biomass load, moisture content, density and leaf SA/V ratio all contributed highly to predicted fire spread rate when taking phylogeny into account (Table 3). Fuel load contributed directly to reaction intensity and indirectly to the propagating flux ratio, via bulk density. Biomass moisture content contributed to spread rate by increasing the heat required for ignition and damping the reaction intensity (see Fig. S2). Leaf SA/V ratio influenced reaction intensity and the proportion of this reaching adjacent fuel (propagating flux ratio), as well as the proportion of fuel raised to ignition temperature (effective heating number; Fig. S2). Leaf EHoC contributed to the reaction intensity but played a small part in determining the overall predicted rate of spread (Table 3; Fig. S2).

### Trait Covariance

**Figure 3:**
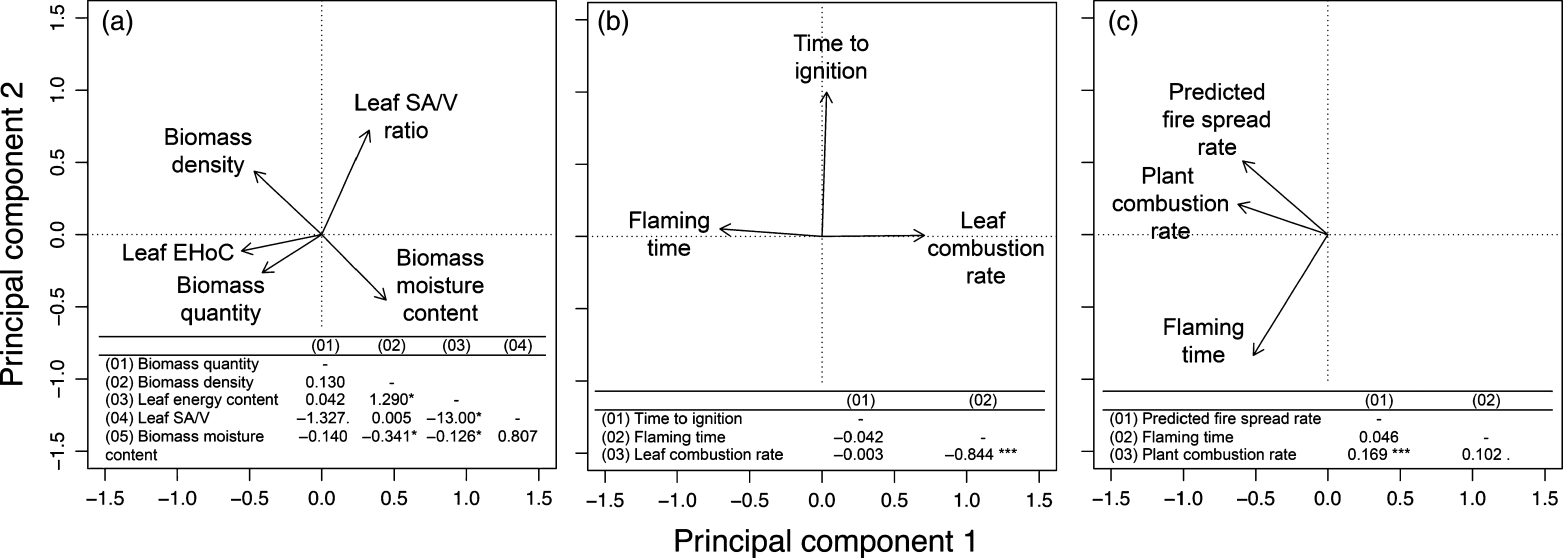
Principal components analysis biplots of explanatory plant traits (a) and flammability traits at the leaf scale (b) and plant scale (c). The tables within each plot indicate the slope and significance of linear regressions between each pair of variables. Data for all traits were log‐transformed to improve normality except leaf SA/V ratio. EHoC is the leaf effective heat of combustion. *P *<* *0.1; *, *P *<* *0.05; ***, *P *<* *0.001.

Principal components analysis (PCA) and linear regressions were used to explore patterns of covariance among the plant and flammability trait variables, with the latter being split into leaf‐scale and plant‐scale traits (Fig. 3). For the plant traits, the first two principal components accounted for 67.6% of the total variance. The first axis related to the chemical properties of biomass and how it is arranged spatially (leaf EHoC, biomass moisture content and density had the highest axis loadings). Leaf SA/V ratio loaded most heavily on the second axis, followed by biomass moisture content and density. Only biomass quantity did not fall as clearly on the first two principal components, which we believe is due to the high variation within the data (CV = 89.0%). For the leaf‐scale flammability traits, the first two principal components accounted for 95.1% of the total variance. Leaf flaming time and combustion rate were negatively correlated (*P *<* *0.001), and fell in opposing directions on the first PCA axis (Fig. 3), which reflects how combustion rate was derived from flaming time. Time to ignition was unrelated to flaming time and combustion rate and was orthogonal to both in the PCA (Fig. 3). For plant‐scale flammability traits, 71.8% of total variance is accounted for by the first two principal components. Traits did not separate on the first axis, but did on the second axis which related to burning intensity. High rates of plant combustion were associated with rapid predicted fire spread rates (*P *<* *0.001) and marginally with longer flaming times (*P *=* *0.071; Fig. 3).

The relationships between flammability traits measured at different scales were variable, with a significantly positive correlation found for ignitability (leaf time to ignition vs. predicted rate of spread; *P *=* *0.025), but no significant correlation for combustibility (leaf‐scale combustion rate vs. plant‐scale combustion rate; *P *=* *0.29).

### Influence of Evolutionary History on Flammability

Support for a phylogenetic signal was found for leaf‐scale combustibility (Pagel's λ = 0.99; *P *=* *0.93 for likelihood ratio test against λ = 1; *P *=* *0.037 against λ = 0) and sustainability (Pagel's λ = 0.45; *P *=* *0.67 against λ = 1; *P *=* *0.011 against λ = 0), but not for the other flammability traits. Of the plant traits, there was a strong phylogenetic signal for leaf SA/V ratio (Pagel's λ = 1.00; *P *=* *1.00 against λ = 1; *P *<* *0.001 against λ = 0), with closely related species tending to have similar values of leaf SA/V ratio. No phylogenetic signal was found in the other plant traits.

## Discussion

This large comparative study of grass flammability provides strong support for the hypothesis that grass species vary significantly in multiple components of flammability. This finding suggests that static classifications of grassy and herbaceous vegetation as homogenous fuels mask considerable interspecific and community variation in flammability. Consequently, fire behaviour predictions based on such fuel models may lose accuracy when community composition is not accounted for.

A substantial proportion of variation in ignitability and combustibility (70% and 58%, respectively) can be explained by a combination of the five plant traits measured here. For sustainability, a smaller proportion of variation was accounted for (37%), perhaps because this component is not only driven by plant traits, but is also directly influenced by combustibility. Additionally, some variation in sustainability could be accounted for by traits relating to leaf chemistry, such as nitrogen, phosphorus and tannin concentrations (Grootemaat *et al*. 2015) that were not measured in this study. Biomass quantity was the key trait influencing plant‐scale flammability components and also determined the influence of an individual plant on local fire characteristics. The importance of biomass quantity for combustibility, sustainability and fire spread rates in the field is illustrated by the elevated flammability of landscapes caused by the raised fuel load production of non‐native grasses (Hughes, Vitousek & Tunison 1991; D'Antonio & Vitousek 1992; Rossiter *et al*. 2003). While making a relatively small contribution to flammability components once alight, biomass moisture content was key to ignitability, with higher moisture contents requiring more energy to dry and heat biomass to the point of ignition (Trollope 1978; Gill & Moore 1996; Alessio *et al*. 2008; Plucinski & Anderson 2008). By influencing ignitability, and therefore the likelihood of fire occurring in the first place, moisture content exerts a strong influence on vegetation flammability. Our finding of high interspecific variation in EHoC (effective heat of combustion) also conflicts with the notion that grass energy content is an almost constant value (Trollope 1984). However, EHoC contributed little to leaf‐scale flammability components, supporting the idea that this intrinsic property is less important in determining flammability than fuel mass, structure and moisture content (Bond & van Wilgen 1996). Despite this small importance overall, the EHoC marginally contributed to plant‐scale flaming time.

The inconsistent relationships between components of flammability, and within flammability components measured at different scales, suggest that descriptions of flammability should incorporate all suitable components and should be taken at an appropriate scale. The mixed covariance between flammability components found here suggests that one cannot always be used as a proxy for the others. Therefore, studies that consider one or even two components of flammability may mask the complexity of vegetation flammability (Anderson 1970). Similar to the findings of Martin (2010), we find support for the importance of incorporating plant architecture into measurements of grass flammability. Inconsistencies between combustibility at the leaf‐ and plant‐scale highlight that other factors (such as biomass quantity and density) are key determinants of combustibility at the plant scale. Bench‐scale measurements of flammability have been criticized as not being representative of flammability in the field (Fernandes & Cruz 2012), and our findings emphasize the need for caution when extrapolating flammability traits between different scales. In comparison with leaf‐scale studies, the flammability component values obtained here are more representative of flammability in the field because they are measured at the plant scale and on field‐state plants that are at the phenological stage most appropriate to fire occurrence.

The phylogenetic signal found in some flammability components (leaf‐scale combustibility and sustainability) suggests that evolutionary history may partially explain patterns of grass flammability and the strong sorting of grass lineages across fire‐frequency gradients (Uys, Bond & Everson 2004; Visser *et al*. 2012; Forrestel, Donoghue & Smith 2014). However, conclusions on phylogenetic signal derived from a small phylogeny must remain cautious due to low statistical power (Boettiger, Coop & Ralph 2012).

Through their flammability, plants may modify the fire regime they experience in order to increase their fitness in fire‐prone environments (Schwilk 2003). Resprouting grasses are likely to benefit from frequent fires that remove standing biomass and maintain an open canopy, because they are typically intolerant of shading (Everson, Everson & Tainton 1988; Bond 2008). The grasses studied here showed high ignitability, combustibility and predicted fire spread rates, when compared to woody vegetation fuels (e.g. Pausas *et al*. 2012; Ganteaume *et al*. 2013). Furthermore, grasses are able to regrow quickly after fire. This combination of high flammability and rapid regrowth drives a fire regime characterized by high fire frequency (Grigulis *et al*. 2005). Plant‐scale combustion rate was marginally positively related to flaming time, with high biomass plants burning at a faster rate and for longer. This finding is in contrast with other studies (e.g. de Magalhães & Schwilk 2012) that found a negative relationship between the two. It also does conflicts with the idea of high flammability providing resprouting plants protection against lethal temperatures (Gagnon *et al*. 2010), as for grasses that have higher fuel loads, rapid combustion is not associated with lowered burning durations and a subsequent reduction in heat transfer to the soil and below‐ground plant parts. The interspecific variation in flammability components observed across a set of species that commonly coexist in the field further suggests a role for interspecific competition in promoting flammability as an adaptive trait. Potentially, enhanced plant flammability can increase the mortality of neighbouring, less fire‐tolerant individuals and thereby reduce post‐fire competition (Bond & Midgley 1995). Furthermore, some evidence provides intriguing support for a link between high flammability and ecological success in fire‐prone grassland species (Ripley *et al*. 2015). The influence of flammability at the species level on grassland community‐level flammability has not been determined. However, findings from other vegetation fuel types show that flammability tends to be driven by the most flammable species of a community, such that fuel loads are non‐additive (van Altena *et al*. 2012; de Magalhães & Schwilk 2012). The knowledge gained in this study can be used in further work to determine whether high flammability is an adaptation to life in frequently burnt environments for grasses and has thus been a fundamental trait in grass evolution. In addition, the knowledge of interspecific variation in grass flammability obtained here can lead to a better understanding of wildfire behaviour, particularly in grassland ecosystems. This could potentially contribute to an improvement of global carbon modelling and lead to new insights about ecosystem feedback to fire.

## Data accessibility

Trait data: Species average values uploaded as online supporting information; raw data available in DRYAD entry doi: 10.5061/dryad.2c506.

Sequence data: GenBank accession numbers available as online supporting information.

Phylogeny: Nexus file available in DRYAD entry doi: 10.5061/dryad.2c506.

MCMCglmm R Script: Available in DRYAD entry doi: 10.5061/dryad.2c506.

## Supporting information


**Figure S1.** Schematic drawing of the set‐up used to measure plant‐scale combustibility and sustainability.
**Figure S2.** Cumulative dry biomass over vertical plant height for the grass species.
**Figure S3.** The influence of plant traits on components of Rothermel's (1972) fire spread rate model.

**Table S1.** Climate data from plant collection sites.
**Table S2.** Grass species names, collection site and GenBank accession details.
**Table S3.** Plant traits values used to model the forward rate of fire spread (m min^−1^).
**Table S4.** Species mean flammability component values.
**Table S5.** Species mean plant trait values.
**Table S6.** Results of analysis of variance (two‐way anova with interaction) of leaf‐scale flammability by species and state (fresh or dry).
**Table S7.** Mean plant trait values for the three collection sites.
