# Supplementary material for: Determinants of flammability in savanna grass species
Source: J Ecol. 2015 Nov 26;104(1):138–48. doi: 10.1111/1365-2745.12503 (PMC4738432; doi:10.1111/1365-2745.12503)
Supplement: Supplementary file 1 — Figure S1. Schematic drawing of the set‐up used to measure plant‐scale combustibility and sustainability. Figure S2. Cumulative dry biomass over vertical plant height for the grass species. Figure S3. The influence of plant traits on components of Rothermel's (1972) fire spread rate model. [file JEC-104-138-s001.docx]

*Supporting Information - Figures*

**
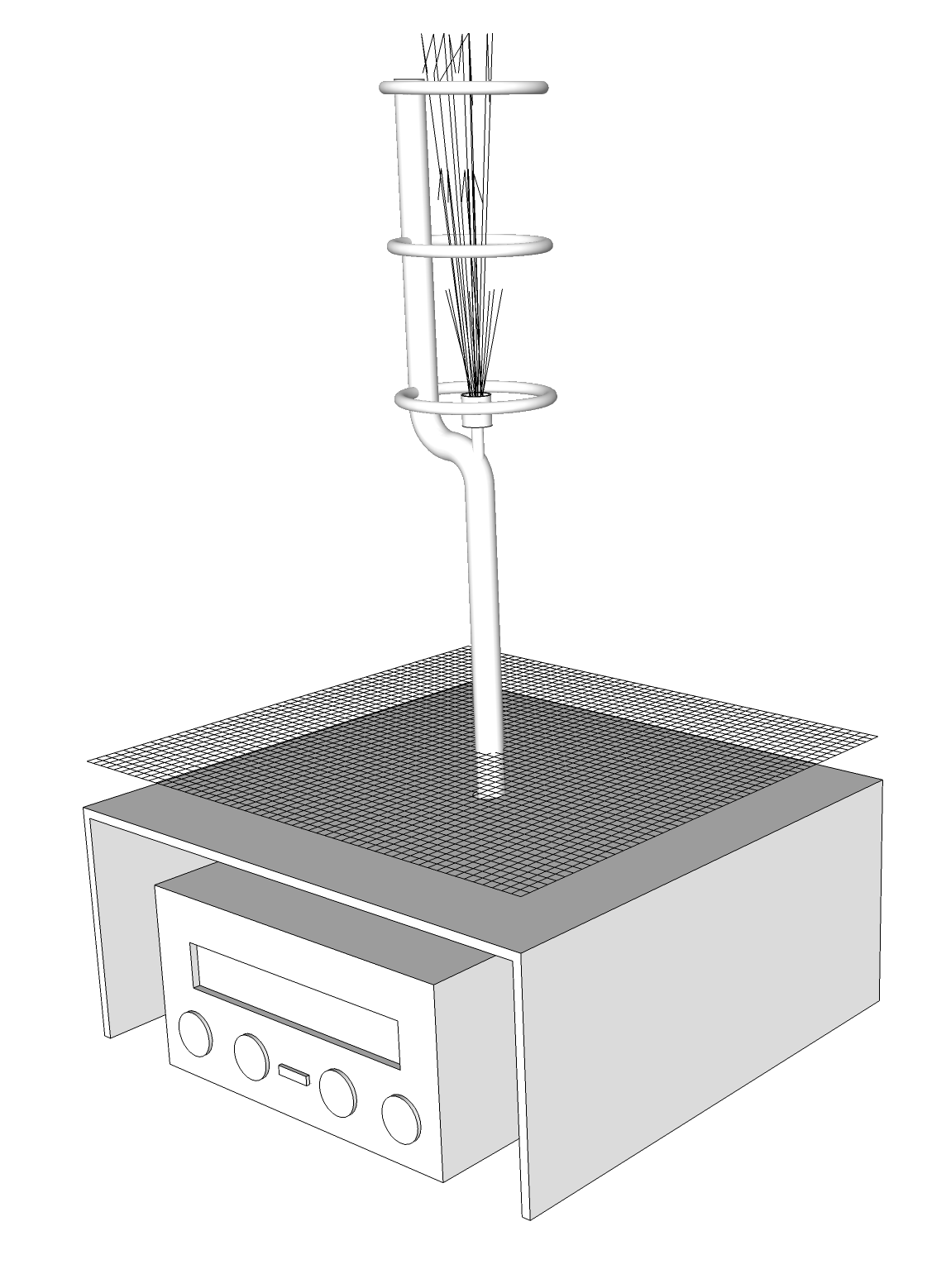
**

(f)

(a)

(e)

(b)

(d)

(c)

**Figure S1.** Schematic drawing of the set-up used to measure plant-scale combustibility and sustainability. (a) Clamp for holding grass material; (b) mesh platform for catching falling biomass; (c) four point balance; (d) wooden box (to protect the scales from heat damage); (e) stand; (f) metal wire cage (to prevent biomass from falling off). Samples were ignited by directing a bunsen burner flame to the side of the base of the clump at a 45° angle and a 5cm distance for 3 seconds maximum (less if ignition happened earlier).

**

**Figure S2.** Cumulative dry biomass over vertical plant height for 25 grass species. Each line represents an individual (n=7 per species). Both axes were log-transformed in order to fit a linear model to the data for each individual. Species name abbreviations: A. =*Alloteropsis*; Ar.c.=*Aristida congesta*; C.=*Cenchrus*; Cy.=*Cymbopogon*; Cyn.=*Cynodon*; D.=*Digitaria*; E.=*Eragrostis*; Eu.=*Eustachys*; M.=*Melica*; Me.=*Melinis*; Mer.=*Merxmuellera*; P.=*Panicum*; S.=*Setaria*; Sp.=*Sporobolus*; T.=*Themeda*; Tr.=*Tristachya*.

**Figure S3**. The influence of plant traits on components of Rothermel’s (1972) fire spread rate model. The flow chart demonstrates how the input plant traits determine the fire spread components. Graphs *i-iv* show relationships between plant traits and components of the fire spread equation. Points reflect trait values obtained from individual plants, and the lines are the modelled relationships between plant trait and fire spread component.

*i*. The relationship between plant SA/V ratio (σ) and maximum reaction velocity (γ_max_)

*ii*. The relationship between biomass moisture content (M_f_) and the moisture damping coefficient ($n_{M}$)

*iii*. The relationship between plant SA/V ratio (σ) and the propagating flux ratio (ξ, the proportion of reaction intensity reaching adjacent fuel), taking into account the influence of wind (1 + Φw). Line reflects the relationship between σ and ξ (1 + Φw) when values of δ (fuel height) and W_0_ (oven dry fuel load) are held constant.

*iv*. The relationship between plant SA /V ratio (σ) and the effective heating number (ε; the proportion of fuel that is raised to ignition temperature).

Equations

$$n_{M}=1-2.59\left( \frac{M_{f}}{M_{x}} \right)+\left( \frac{M_{f}}{M_{x}} \right)^{2}-3.52\left( \frac{M_{f}}{M_{x}} \right)^{3}$$

Where: M_x_ = fuel moisture content of extinction (= 0.6)

$$Pb=\frac{1}{\delta}W_{0}$$

$$\beta=\frac{Pb}{Pp}$$

Where Pp = oven dry particle density (kg m^-3^)

$$\gamma_{max}=\sigma^{1.5}\left( 495+0.0594\sigma^{1.5} \right)^{-1}$$

$$Q_{ig}=250+1116M_{f}$$

$$\varepsilon=exp(\frac{-138}{\sigma})$$

$$\Phi_{W}=CU^{B}\left( \frac{\beta}{\beta_{op}} \right)^{-E}$$

Where: C = 7.47 exp(-0.133σ^0.55^); B = 0.02526σ^0.54^; E = 0.715 exp(-3.59 x 10^-4^σ);

U = midflame wind speed ; βop = 3.348σ^-0.8189^

$$\xi=\left( 192+0.2595\sigma\right)^{-1}exp[\left( 0.792+0.681\sigma^{0.5} \right)\left( \beta+0.1 \right)$$

$$I_{R}=\gamma maxW_{n}Hn_{M}n_{s}$$

Where: n_S_ = mineral damping coefficient (= 0.41739)

$$R=(I_{R}\xi\left( 1+\Phi_{W}+\Phi_{S} \right))/(PbℇQ_{ig})$$

Where: Φs = slope coefficient ( = 0)
