# Supplementary material for: Determinants of flammability in savanna grass species
Source: J Ecol. 2015 Nov 26;104(1):138–48. doi: 10.1111/1365-2745.12503 (PMC4738432; doi:10.1111/1365-2745.12503)
Supplement: Supplementary file 2 — Table S1. Climate data from plant collection sites. Table S2. Grass species names, collection site and GenBank accession details. Table S3. Plant traits values used to model the forward rate of fire spread (m min−1). Table S4. Species mean flammability component values. Table S5. Species mean plant trait values. Table S6. Results of analysis of variance (two‐way anova with interaction) of leaf‐scale flammability by species and state (fresh or dry). Table S7. Mean plant trait values for the three collection sites. [file JEC-104-138-s002.docx]

| Site name | Coordinates (lat, lon) | Average July daily wind speed  (m s^-1^) | Total July daily rain (mm) | Minimum July daily temperature (°C) | Maximum July daily temperature (°C) |
| --- | --- | --- | --- | --- | --- |
| Site 1 | -33.323, 26.533 | 3.5 | 27.8 | 6.8 | 18.5 |
| Site 2 | -33.278, 26.489 | 3.5 | 27.8 | 6.8 | 18.6 |
| Site 3 | -32.965, 26.082 | 2.2 | 0.1 | 7.6 | 19.6 |

*Supporting Information - Tables*

**Table S1.** Climate data from plant collection sites including wind speed, rainfall and temperature. For sites 1 and 2, average wind speed, rainfall and temperature values for July (i.e. the month in which collection occurred) were provided by the South African Weather Service (weather station 0056917 8; www.weathersa.co.za) for years spanning 1993 to 2014. Data for site 3 was provided by the Agricultural Research Council (www.arc.agric.za) and is for years 2012 and 2013

**Table S2.** Grass (Poaceae) species used in this study, collection site and GenBank accession number.

| Species | Subfamily | Site | GenBank accession number |
| --- | --- | --- | --- |
| *Alloteropsis semialata* (R.Br.) subsp. *eckloniana* | Panicoideae (Paniceae) | 1 | (See Grass Phylogeny Working Group II 2012) |
| *Aristida congesta* Roemer & Schult subsp*. barbicollis* (Trin & Rupr.) De Winter | Aristidoideae | 3 | KP860326 |
| *Cenchrus setaceus* (Forssk.) Morrone | Panicoideae (Paniceae) | 2 | (See Grass Phylogeny Working Group II 2012) |
| *Cymbopogon plurinodis* (K. Schum.) C.E. Hubb. | Panicoideae (Andropogonea) | 3 | KP860328 |
| *Cymbopogon nardus* (L.) Rendle. | Panicoideae (Andropogonea) | 1 | KP860327 |
| *Cynodon dactylon* (L.) Pers. | Chloridoideae | 1 | (See Grass Phylogeny Working Group II 2012) |
| *Digitaria eriantha* Steud. | Panicoideae (Paniceae) | 2 | (See Grass Phylogeny Working Group II 2012) |
| *Eragrostis curvula* (Schrad.) | Chloridoideae | 1 | (See Grass Phylogeny Working Group II 2012) |
| *Eragrostis lehmanniana* Nees | Chloridoideae | 3 | KP860329 |
| *Eragrostis plana* Nees | Chloridoideae | 2 | KP860330 |
| *Eustachys paspaloides* (Vahl) Lanza & Mattei | Chloridoideae | 3 | KP860331 |
| *Heteropogon contortus* (L.) | Panicoideae (Andropogonea) | 1 | (See Grass Phylogeny Working Group II 2012) |
| *Hyparrhenia hirta* (L.) Stapf. | Panicoideae (Andropogonea) | 2 | (See Grass Phylogeny Working Group II 2012) |
| *Melica racemosa* Thunb. | Pooideae | 2 | KP860332 |
| *Melinis* sp. | Panicoideae (Paniceae) | 1 | KP860335 |
| *Melinis nerviglumis* (Franch.) Zizka | Panicoideae (Paniceae) | 2 | KP860333 |
| *Merxmuellera disticha* (Nees) Conert | Danthonioideae | 2 | (See Grass Phylogeny Working Group II 2012) |
| *Merxmuellera stricta* (Schrad.) Conert | Danthonioideae | 1 | (See Grass Phylogeny Working Group II 2012) |
| *Panicum aequinerve* Nees | Panicoideae (Paniceae) | 1 | (See Grass Phylogeny Working Group II 2012) |
| *Panicum* sp. | Panicoideae (Paniceae) | 1 | KP860334 |
| *Pentameris* sp. | Danthonioideae | 1 | KP860336 |
| *Setaria sphacelata* (Schumach.) Stapf & C.E. Hubb. Ex. M.B.Moss | Panicoideae (Paniceae) | 3 | (See Grass Phylogeny Working Group II 2012) |
| *Sporobolus indicus* (L.) R.Br. | Chloridoideae | 2 | (See Grass Phylogeny Working Group II 2012) |
| *Themeda triandra* Forssk. | Panicoideae (Andropogonea) | 1 | (See Grass Phylogeny Working Group II 2012) |
| *Tristachya leucothrix* Trin ex Nees. | Panicoideae (Paniceae) | 1 | (See Grass Phylogeny Working Group II 2012) |

**Table S3.** Plant traits values input to obtain forward rate of fire spread (m min^-1^). Spread rate values were calculated for each individual by creating surface fire spread models (Rothermel 1972) based on the plant traits of each individual. The models assumed uniform weather, topography and fuel for the time duration. Biomass density could not be input directly but was calculated as fuel load divided by plant height. All individuals were considered as ‘1-hour fuels’

| Model parameter | Method of calculation |
| --- | --- |
| Fuel load (t ha^-1^) | Dry total plant biomass and plant width values were used to calculate a value of biomass per unit area for each individual. Plant width (the maximum spread of photosynthetic material parallel to the soil level) was averaged per species and converted into an area (using area=π(width/2)^2^), and the total fresh plant biomass was divided by this. Plant width values were standardised at the species level as small intra-specific variation in this trait had considerable impacts on individual fuel load values. |
| Fuel SA/ Volume ratio (m^2^/m^3^) | Leaf SA/V values. |
| Fuel bed depth (cm) | Plant height values. |
| Dead fuel moisture of extinction (%) | This parameter is the characteristic moisture content of a dead fuel above which a steady rate of fire spread is not possible. A value of 60% was used for all models as this is the maximum possible for 1-hour fuels. |
| Fuel heat content  (kJ kg^-1^) | Species average effective heat of combustion (EHoC) values were used. As these values fell below the range accepted by the model, all were multiplied by the lowest factor necessary (1.93) to put them into the acceptable range of values. Preliminary tests showed a linear relationship between rate of spread and fuel heat content so multiplying all values by a common factor was deemed acceptable. |
| Fuel moisture content (%) | Biomass moisture content values |
| Midflame wind speed (km h^-1^) | An average value of July daily wind speed for the three collection sites was used in all models (3 m s^-1^ = 3.6 km h^-1^). |
| Site slope (%) | A value of 0 was used for all models. |

**Table S4.** Flammability parameter values (mean ± standard error) for 25 grass species, and the influence of species on these values. The final two rows indicate F values obtained from ANOVA and the degrees of freedom (DF) for all flammability parameters except plant combustion rate. The data for this trait precluded the use of statistical testing because there was only one value per species. ***, P<0.001.

| Species | Time to ignition (s) | Flaming time  (s) | Leaf combustion rate (mg s^-1^) | Plant combustion rate (mg s^-1^) | Fire spread rate  (m min^-1^) |
| --- | --- | --- | --- | --- | --- |
| *Alloteropsis*  *semialata* subsp. *eckloniana* | 2.19±0.22 | 7.13±3.19 | 29.0±2.2 | 94.0±22.0 | 1.55± 0.123 |
| *Aristida congesta*  subsp*. barbicollis* | 1.75±0.44 | 4.36±1.65 | 49.0±5.0 | 97.0±16.0 | 2.48± 0.252 |
| *Cenchrus*  *setaceus* | 3.96±1.07 | 6.02±3.01 | 39.0±10.1 | 74.0±9.0 | 0.00±0.000 |
| *Cymbopogon*  *plurinodis* | 1.93±0.16 | 6.32±2.39 | 33.0±2.2 | 67.0±12.0 | 0.73±0.173 |
| *Cymbopogon*  *nardus* | 1.23±0.11 | 5.51±2.08 | 37.0±2.1 | 86.0±18.0 | 5.34±0.310 |
| *Cynodon dactylon* | 1.34±0.18 | 6.26±2.36 | 32.0±0.6 | 121.0±25.0 | 3.36±0.218 |
| *Digitaria eriantha* | 1.10±0.07 | 5.31±2.01 | 41.0±4.4 | 122.0±28.0 | 2.25±0.482 |
| *Eragrostis curvula* | 1.40±0.14 | 6.17±2.33 | 34.0±2.6 | 70.0±12.0 | 2.94±0.406 |
| *Eragrostis*  *lehmanniana* | 1.62±0.15 | 6.34±2.40 | 32.0±2.0 | 71.0±13.0 | 2.76±0.190 |
| *Eragrostis plana* | 2.53±0.53 | 7.68±2.90 | 27.0±1.9 | 81.0±14.0 | 0.53±0.100 |
| *Eustachys*  *paspaloides* | 1.56±0.19 | 5.81±2.20 | 38.0±5.1 | 64.0±19.0 | 0.69±0.158 |
| *Heteropogon*  *contortus* | 1.93±0.20 | 4.73±1.79 | 43.0±2.8 | 132.0±57.0 | 1.80±0.107 |
| *Hyparrhenia hirta* | 1.02±0.08 | 4.95±1.87 | 43.0±3.4 | 124.0±51.0 | 6.37±0.538 |
| *Melica racemosa* | 1.47±0.14 | 5.58±2.11 | 37.0±2.1 | 82.0±10.0 | 1.72±0.210 |
| *Melinis* *nerviglumis* | 1.21±0.13 | 5.91±2.24 | 35.0±2.4 | 137.0±20.0 | 2.04±0.148 |
| *Melinis* sp. | 1.32±0.12 | 6.41±2.42 | 32.0±1.5 | 147.0±23.0 | 1.08±0.154 |
| *Merxmuellera*  *disticha* | 2.13±0.23 | 6.95±2.63 | 29.0±1.3 | 111.0±36.0 | 0.237±0.091 |
| *Merxmuellera*  *stricta* | 1.82±0.26 | 6.55±2.48 | 31.0±1.7 | 113.0±22.0 | 0.42±0.068 |
| *Panicum*  *aequinerve* | 1.20±0.10 | 5.23±1.98 | 38.0±1.1 | 94.0±25.0 | 2.03±0.145 |
| *Panicum* sp. | 1.90±0.23 | 5.62±2.13 | 37.0±3.5 | 93.0±22.0 | 1.32±0.169 |
| *Pentameris* sp. | 1.46±0.17 | 5.47±2.07 | 37.0±2.2 | 86.0±24.0 | 1.09±0.164 |
| *Setaria sphacelata* | 1.38±0.12 | 4.79±1.81 | 44.0±4.3 | 117.0±15.0 | 0.69±0.133 |
| *Sporobolus indicus* | 1.07±0.15 | 6.47±2.64 | 32.0±2.4 | 73.0±10.0 | 0.57±0.058 |
| *Themeda triandra* | 1.13±0.14 | 5.10±1.93 | 42.0±4.4 | 163.0±24.0 | 2.58 ±0.181 |
| *Tristachya leucothrix* | 1.58±0.06 | 5.71±2.16 | 36.0±2.5 | 106.0±29.0 | 2.71±0.324 |
| F value | 5.02 *** | 3.02 *** | 2.97 *** | N/A | 42.42 *** |
| DF | 24, 144 | 24, 144 | 24, 144 | N/A | 24, 150 |

**Table S5.** The influence of species on plant trait values (mean ± standard error) of 25 grass species as indicated by F values obtained from ANOVA. ***, P<0.001.

| Species | Biomass quantity (g) | Biomass density  (g cm^-1^) | Biomass moisture content (g g^-1^) | Leaf SA/ volume ratio | Leaf EHoC  (kJ g^-1^) |
| --- | --- | --- | --- | --- | --- |
| *Alloteropsis*  *semialata* subsp. eckloniana | 32.29±5.78 | 0.690±0.020 | 0.283±0.030 | 1402.2±40.2 | 9.2±0.5 |
| *Aristida congesta*  *subsp. barbicollis* | 0.55±0.04 | 0.304±0.023 | 0.121±0.009 | 1709.2±75.8 | 8.6±0.1 |
| *Cenchrus*  *setaceus* | 15.55±1.69 | 0.194±0.015 | 1.034±0.088 | 1118.3±58.2 | 7.3±0.2 |
| *Cymbopogon*  *nardus* | 22.52±1.56 | 0.452±0.023 | 0.113±0.007 | 1489.2±49.6 | 9.9±0.6 |
| *Cymbopogon*  *plurinodis* | 1.77±0.25 | 0.267±0.024 | 0.386±0.047 | 1378.1±58.0 | 8.7±0.1 |
| *Cynodon dactylon* | 3.81±0.46 | 0.762±0.040 | 0.104±0.005 | 1729.6±62.7 | 11.4±0.1 |
| *Digitaria eriantha* | 6.23±0.53 | 0.386±0.024 | 0.296±0.052 | 1869.9±76.6 | 8.7±0.2 |
| *Eragrostis curvula* | 18.05±2.31 | 0.391±0.004 | 0.113±0.018 | 1135.5±60.6 | 8.9±0.3 |
| *Eragrostis*  *lehmanniana* | 7.84±0.51 | 0.170±0.021 | 0.135±0.013 | 1292.8±71.7 | 8.8±0.1 |
| *Eragrostis plana* | 1.51±0.20 | 0.309±0.028 | 0.379±0.039 | 989.6±82.1 | 10.1±0.2 |
| *Eustachys*  *paspaloides* | 1.55±0.41 | 0.259±0.032 | 0.356±0.034 | 1599.2±60.3 | 7.7±0.6 |
| *Heteropogon*  *contortus* | 10.52±1.73 | 0.378±0.027 | 0.242±0.020 | 1579.3±93.3 | 11.0±0.3 |
| *Hyparrhenia hirta* | 16.85±3.82 | 0.500±0.023 | 0.158±0.019 | 1743.9±80.7 | 8.2±0.4 |
| *Melica racemosa* | 5.25±1.49 | 0.733±0.046 | 0.182±0.033 | 938.7±27.5 | 10.3±0.5 |
| *Melinis* *nerviglumis* | 5.59±0.50 | 0.436±0.028 | 0.137±0.008 | 1045.9±52.8 | 10.1±0.5 |
| *Melinis* sp. | 9.15±1.11 | 0.362±0.027 | 0.127±0.011 | 961.2±43.0 | 12.4±0.2 |
| *Merxmuellera*  *disticha* | 27.75±6.24 | 0.479±0.037 | 0.115±0.010 | 386.0±14.9 | 10.9±0.3 |
| *Merxmuellera*  *stricta* | 22.31±2.55 | 0.792±0.035 | 0.110±0.012 | 564.6±22.3 | 10.7±0.6 |
| *Panicum*  *aequinerve* | 3.06±0.34 | 0.735±0.034 | 0.260±0.024 | 1864.3±82.3 | 9.6±0.3 |
| *Panicum* sp | 6.67±0.69 | 0.369±0.031 | 0.297±0.041 | 1058.7±99.8 | 9.1±0.5 |
| *Pentameris* sp. | 5.89±0.68 | 0.435±0.026 | 0.158±0.008 | 871.2±45.7 | 11.5±0.6 |
| *Setaria sphacelata* | 1.21±0.18 | 0.362±0.039 | 0.386±0.034 | 1597.1±78.7 | 8.0±0.1 |
| *Sporobolus indicus* | 4.50±0.54 | 0.306±0.023 | 0.411±0.030 | 906.1±70.2 | 11.2±0.2 |
| *Themeda triandra* | 8.37±1.34 | 0.753±0.034 | 0.167±0.010 | 2078.2±54.7 | 8.8±0.0 |
| *Tristachya leucothrix* | 28.07±4.88 | 0.651±0.027 | 0.165±0.013 | 1065.0±58.8 | 13.6±0.4 |
| *F_24,150_ value* | 16.25 *** | 18.09 *** | 39.75 *** | 44.38 *** | 17.38 *** |

**Table S6.** Results of analysis of variance (two-way ANOVA with interaction) of leaf scale flammability by species and state (fresh or dry). P values in bold are significant at P=0.05. DF= degrees of freedom

| Flammability trait | Source | Df | F | p |
| --- | --- | --- | --- | --- |
| Time to ignition (s) | State | 1 | 52.42 | **<0.001** |
|  | Species | 24 | 5.930 | **<0.001** |
|  | State*Species | 24 | 3.650 | **<0.001** |
| Flaming time (s) | State | 1 | 6.147 | **0.014** |
|  | Species | 24 | 6.894 | **<0.001** |
|  | State*Species | 24 | 0.641 | 0.904 |
| Combustion rate (g s^-1^) | State | 1 | 7.584 | **0.006** |
|  | Species | 24 | 7.289 | **<0.001** |
|  | State*Species | 24 | 0.648 | 0.919 |

**Table S7.** Mean plant trait values of the three collection sites. Traits that were significantly influenced by site (One way ANOVA; p<0.05) were subjected to a post hoc pairwise test (Tukey HSD). Superscripted letters reflect the significance of pairwise comparisons with different letters signifying significant differences (p<0.05). SD = Standard deviation, n = sample size (number of species)

|  |  | Mean site values ± SD | | | | |
| --- | --- | --- | --- | --- | --- | --- |
| Site | n | Average leaf EHoC  (kJ g^-1^) | Average leaf SA/Volume ratio | Average aboveground dry biomass (g) | Average Biomass water content  (g g^-1^) | Average vertical biomass distribution  (g cm^-1^) |
| 1 | 12 | 10.31±1.38^a^ | 13.24±4.43 | 13.93±10.22 | 0.179±0.071 | 0.566± 0.183^a^ |
| 2 | 8 | 9.87±1.71^ab^ | 11.14±4.80 | 10.85±8.68 | 0.338±0.303 | 0.420±0.161^ab^ |
| 3 | 5 | 8.34±0.49^b^ | 15.15±1.73 | 2.58±2.97 | 0.277±0.137 | 0.277±0.078^b^ |
